# Zebrafish as a Model to Study Retinoic Acid Signaling in Development and Disease

**DOI:** 10.3390/biomedicines11041180

**Published:** 2023-04-15

**Authors:** Matthew R. Hawkins, Rebecca A. Wingert

**Affiliations:** Department of Biological Sciences, Center for Stem Cells and Regenerative Medicine, Center for Zebrafish Research, Boler-Parseghian Center for Rare and Neglected Diseases, Warren Center for Drug Discovery, University of Notre Dame, Notre Dame, IN 46556, USA; mhawkin7@nd.edu

**Keywords:** retinoic acid, vitamin A, development, zebrafish, neurogenesis, kidney, heart, endoderm, cancer, regeneration

## Abstract

Retinoic acid (RA) is a metabolite of vitamin A (retinol) that plays various roles in development to influence differentiation, patterning, and organogenesis. RA also serves as a crucial homeostatic regulator in adult tissues. The role of RA and its associated pathways are well conserved from zebrafish to humans in both development and disease. This makes the zebrafish a natural model for further interrogation into the functions of RA and RA-associated maladies for the sake of basic research, as well as human health. In this review, we explore both foundational and recent studies using zebrafish as a translational model for investigating RA from the molecular to the organismal scale.

## 1. Introduction

For nearly 100 years, vitamin A in the maternal diet has been linked to normal embryonic ontogeny in vertebrates. Initially, this was based on observations in female pigs and rats that subsistence on nourishments lacking vitamin A during pregnancy was associated with a plethora of birth defects in newborns ranging from eye abnormalities to genitourinary defects [1,2,3,4,5,6,7,8]. Continued nutrition research uncovered a complex spectrum of congenital malformations that occurred consequent to a maternal vitamin A deficient (VAD) diet, which came to be known as the VAD syndrome [9]. The malformations included defects in the central nervous system, eyes, ears, heart, lungs, limbs, skin, and urogenital system [9]. These and subsequent studies helped to stimulate continued research on how retinoids, the biologically active metabolites of vitamin A, modulate development. Many fundamental insights were uncovered by investigating the teratogenic effects of RA—how global or local exposure impacted normal processes [10,11,12,13,14,15,16]. RA signaling is now appreciated as being essential for the genesis of nearly every vertebrate tissue and organ [10,11,12,13,14,15,16]. The wide range of RA functions include patterning of the body axis, regional patterning of the central nervous system, neurogenesis, limb development, and pleiotropic roles in organogenesis [10,11,12,13,14,15,16].

The zebrafish, *Danio rerio*, is a powerful experimental model for studying the roles of RA signaling during ontogeny [17] due to its ex utero development, large embryonic size, and transparent nature—features which have facilitated classical and chemical genetic approaches [18,19]. Here, we provide an overview of RA biology and the zebrafish model, and discuss how zebrafish have been used to further our understanding about the roles of RA during embryogenesis of the neural plate, kidney, heart, blood, endoderm, and in disease states like cancer.

## 2. RA and Zebrafish, a Background

### 2.1. Evolutionary Perspective and Gene Regulation

RA-mediated morphogenic signaling was once thought to represent an evolutionary split between chordates and non-chordates, as RA is necessary for the anterior-posterior (AP) axis and later germ layer organization in early development [20]. However, analysis involving both genomic and proteomic methods has highlighted evidence that traces essential RA-related machinery to far more ancient phylogenies, such as mollusks [20]. Even within chordates, machinery slightly differs along the evolutionary timeline. Within tunicates and other invertebrate chordates, the number of RA receptors is markedly lower due to the two whole genome duplications associated with vertebrate evolution [21]. Despite the change in the number of receptors, the importance of RA in development is still highly conserved across phyla. Tunicates such as *C. intestinalis* treated with exogenous RA were found to have limited neural tube closure, a feature conserved through zebrafish and into humans [22,23,24].

Across species, researchers have linked RA, its receptors, and the accompanying response elements as upstream regulators to homeobox (*hox*) genes, a gene family highly associated with AP positioning, neural crest organization, and overall organogenesis later in development [25,26,27,28]. In the zebrafish genome, *hox* genes are isolated to clusters, and this multiple cluster model is conserved throughout vertebrates [29,30,31]. Though these clustered identities are well conserved, the number of clusters containing protein coding regions does differ. In comparison to mammals which have four of these clusters, zebrafish have seven, the odd number due to a condensation post genome-wide duplication [32,33,34]. Genes within each of these clusters are genetically positioned 3′ to 5′ in the order in which they are transcribed, meaning upstream elements represent coding regions for anterior cell fates [35]. This collinear positioning is also sensitive to RA, as exogenous RA treatments can lead to the initiation of transcription for upstream/anterior elements in locations which normally require posterior *hox* elements, and the opposite for downstream/posterior identities [36,37,38].

Other than *hox* gene regulation, RA has a regulatory footprint in various other signaling pathways that aid in developmental processes. One signaling family that should be duly noted is the Fibroblast Growth Factors (FGF) and the ensuing cascades. FGF works on an opposing gradient to RA in the presomitic mesoderm, among other tissues, and is vital for posteriorizing cell fates in cell populations such as the neural plate [39]. In the presence of exogenous RA or RA agonists, *fgf8* expression within caudal neural plate explants is markedly decreased [39]. In these same experiments performed by Diez el Corral et al. utilizing vitamin-A deficient quail, the researchers found that *fgf8* expression extends to more anterior fates, thus portraying RA as a negative regulator of FGFs [39]. In the converse manner, FGFs have the ability to increase RA signaling via *cyp26* suppression. Through Cyp26 enzymes, *fgf8* transcription works to create a margin between the anterior and posterior neural ectoderm in the earliest stages of gastrulation, thus allowing for early organization for future cell fates [40]. RA has a regulatory role on FGF signaling in other developmental processes as well, for example, in defining the cardiac and forelimb progenitor fields [10,11,12,13,14,15,16,17].

### 2.2. Biogenesis, Metabolism, and Receptors Involved

**Figure 1:**
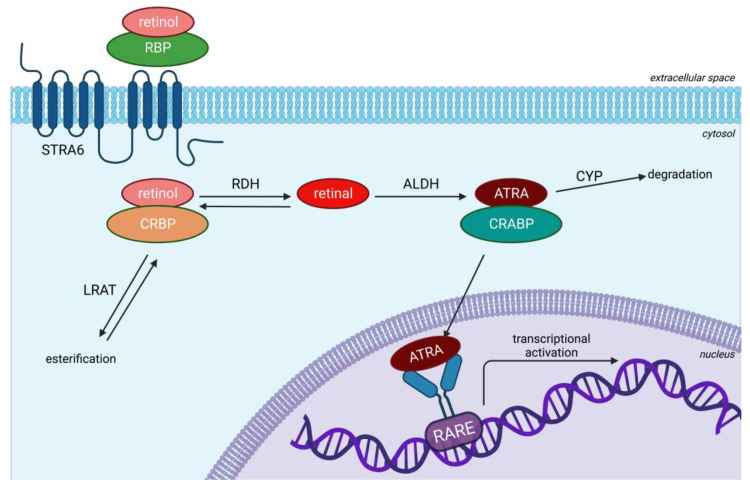
Overview of RA biosynthesis and the canonical pathway of transcriptional activation. Schematic provides a view of the cell membrane, with flanking extracellular space (white) and the cytosol (blue), the latter containing the nuclear compartment (purple). Retinol enters the cell via STRA6(l) proteins, and is bound by CRBP. Next, it can be reversibly esterified for storage by LRAT, or processed by two sequential oxidation reactions by RDH and ALDH enzymes to form ATRA. ATRA can be bound and enter the nucleus upon interactions with RAR/RXR nuclear receptors, or degraded via the action of CYP26 enzymes. Figure created with BioRender.com.

In zebrafish and other vertebrates, de novo RA synthesis does not occur, thus these species rely upon dietary intake of vitamin A or vitamin A preceptors for canonical RA signaling (Figure 1). Forms of vitamin A are present endogenously in the early embryo from maternal sources, e.g., transferred across the placental circulation in mammals or deposited within the egg yolk as carotenoid stores in oviparous species [41,42,43,44,45]. The forms include retinol, retinyl esters, and plant-derived provitamin A carotenoids like β-carotene and β-cryptoxanthin [41,42,43,44,45]. These plant-derived carotenoids must be converted to retinoids via oxygenases such as ββ-carotene-15,15-oxygenase (*bcox*) [46]. In zebrafish, knockdown of the *bcox* gene leads to phenotypes seen in VAD animals; thus, plant-derived carotenoids and their corresponding reducing enzymes are essential for proper development [46].

Once retinol has entered either the serum or the extracellular matrix, retinol binding proteins (RBPs) bind these free retinoids for escort to the cell membrane (Figure 1). This method of transport is found not only in the serum, but in the yolk and the yolk syncytial layer in early development. These bound RBPs act as ligands for the transmembrane transporters of retinol, STRA6 and STRA6l (stimulated by retinoic acid 6, stimulated by retinoic acid 6 like, formerly RBPR2) (Figure 1) [47,48,49,50]. STRA6/l serve as bidirectional gatekeepers of retinol, as RBPs bind to the extracellular facing domain, and cellular retinol binding proteins (CRBPs) bind the cytoplasmic domain [51,52].

Once retinol has bound CRBPs within the cytosol, one of two fates occurs (Figure 1) [11,12,13]. In the presence of lecithin-retinol acyltransferase (LRAT), CRBPII complexes are responsible for the transformations of retinol to retinyl esters [51,52]. These retinyl esters are a stable storage molecule that can be reduced back to retinol in times of RA deficiency [53]. The second fate for CRBP-bound retinol is the oxidation to retinaldehyde via retinol or alcohol dehydrogenases (RDHs or ADHs) (Figure 1) [54]. Once the retinal has been synthesized, the last oxidation of retinaldehyde to RA is facilitated by retinaldehyde dehydrogenases (RALDH, also known as ALDHs) [55,56].

Though several (R)ALDHs are sensitive to retinol, we wish to highlight the (R)ALDH1A family and the three proteins that reside within, two of which are present in zebrafish and other teleosts: *aldh1a2* and *aldh1a3* [57]. In humans, ALDH1A1 is not associated with development; rather, the enzyme has a role in alcohol metabolism and has been linked to several cancers [58]. In zebrafish, *aldh1a2* represents the best described ALDH responsible for RA signaling in development. In the loss of *aldh1a2*, phenotypes in zebrafish development mimic those of VAD embryos in zebrafish and mammals [59,60,61,62,63,64,65]. For example, the death of neural crest cells at 24 h post fertilization (hpf), untidy *hox* expression patterns, and lack of pectoral fin skeletal structure were noted in the *aldh1a2* null fish [59]. In zebrafish, *aldh1a3* knock down via morpholino results in decreased eye size and overall aberrant eye morphology [66]. This phenotype of poor eye formation is thought to be conserved in humans, as mutations within *aldh1a3* result in anophthalmia [67,68]. Transcriptionally within development, *aldh1a2* and *aldh1a3* expression show little temporal overlap [57,59,62,64,69,70]. In whole embryo qRT-PCR experiments performed by Xi et al., *aldh1a2* expression begins during mid gastrulation and tapers off dramatically during segmentation activities [71]. In other work, *aldh1a3* expression was shown to initiate in early somitogenesis/segmentation [57,69]. This falls nearly in step with the earlier work of Liang et al., who showed *aldh1a3* expression to initiate at 14 hpf [69]. This mild discrepancy highlights the need for updated and further investigation of the regulatory networks involved in RA synthesis machinery through the continued application of effective methods, such as qRT-PCR [72,73], and whole mount in situ hybridization (WISH) [74,75,76,77].

Once synthesized, RA has two direct paths to take (Figure 1). The first of which is to bind to a cellular retinoic acid binding protein (CRABP) [78,79] that facilitates transport into the nucleus to bind an RA receptor (Figure 1) [80]. RA receptors represent a body of nuclear receptor superfamily members [81]. In humans and many other vertebrates, three RARs exist (ɑ, β, ɣ), and these RARs are accompanied by retinoid X receptors (RXRs, also: ɑ, β, ɣ) [81,82,83,84]. The coupling of these two receptors creates a complex that will subsequently bind DNA to regulate gene transcription at discrete sequences known as retinoic acid responsive elements (RAREs) (Figure 1) [85,86,87]. The important distinction between RARs and RXRs is the molecules that target them. RARs bind all-trans RA (ATRA) and *9-cis* RA, while RXRs are solely targeted by the *9-cis* RA isomer. Once the ligand and receptor are bound, a heterodimer forms between the twin receptors. Though it is thought that RARs only dimerize with RXRs, it is known that RXRs possess the ability to form homodimers with non-RARs such as Pparɣ to play roles in other pathways [88,89,90,91]. Interestingly, zebrafish, as a result of genome duplication and subsequent condensation, possess four *RAR*s (two *RARɑ* and two *RARɣ*). Despite the loss of *RARβ* from the zebrafish genome, the corresponding *RXR*s were retained through the condensation, and are active in the regulation of various conserved developmental and regenerative processes [92,93,94,95,96,97,98,99].

Due to the need for RA signaling to occur in a gradient that is discrete in terms of concentration, an equally precise mechanism for RA degradation is a necessity, thus providing a second fate for newly synthesized RA (Figure 1). Primarily, three enzymes belonging to the cytochrome p450 26 subfamily (CYP26A1, CYP26B1, and CYP26C1) are tasked with RA metabolism [71,100,101,102,103,104,105,106,107,108]. These enzymes are crucial to tightly control RA distribution and prevent inappropriate signaling within the embryo [109,110]. Zebrafish similarly possess these three *cyp26* genes (*cyp26a1*, *cyp26b1*, and *cyp26c1*), which are closely conserved with humans [71,100,101,102,103,104,105,106,107,108], and are likewise tightly controlled [111,112]. Adjunct functions of each of these Cyp26 enzymes do exist, however, overlaps in tissue-level spatial expression patterns are present [113]. In the course of development, the genes associated with these RA-metabolizing enzymes are found to be activated before synthesis machinery such as *aldh1a2* and *aldh1a3* is transcribed [71]. This accumulation of Cyp26 enzymes before gastrulation indicates potential priming of progenitor populations before the advent of zygotic RA synthesis [71]. This may also be evidence of a fourth enzyme with potential redundant and compensating retinal oxidizing capabilities in early development that is yet to be fully characterized [113]. The presence of Cyp26 family enzymes is also attributed to creating “RA sinks”, which are regions of tissue that act as collecting areas for diffuse/surplus RA [69,111,112,114,115]. These “sinks” occur in part due to RAREs present in the promoters of the *Cyp26a1* gene expression [116,117,118,119,120], and they contribute to the creation of RA morphogen gradients in the early embryo [121,122,123,124,125,126,127].

### 2.3. Zebrafish as a Model Organism

The zebrafish presents as a natural model for developmental work [128] as well as disease modeling [129]. Zebrafish are non-amniotic vertebrates that are fertilized and develop externally within a transparent chorion [130]. This ontogeny can be witnessed readily from the single-cell stage, allowing for genetic manipulation via microinjection and other embryological approaches as well [131,132,133]. The transparent chorion and translucency of the early embryonic stages also enable the precise timing of stage-directed treatments. Cleavage stages transpire rapidly, occurring within the first six hours of development, followed by gastrulation within the first twelve, then subsequent organogenesis from the germ layers [130]. By 24 hpf, a full body plan exists [130]. Within two to three months, the zebrafish is able to breed.

**Figure 2:**
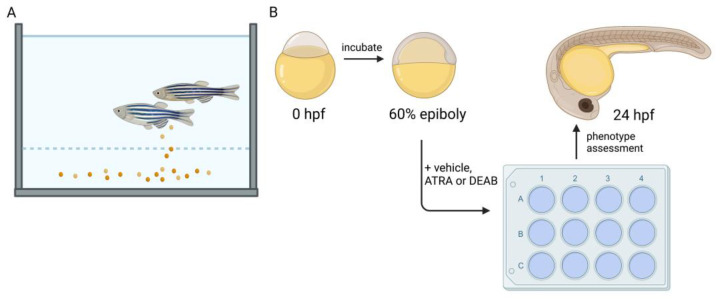
Zebrafish as a model organism. (**A**) Zebrafish adults reach sexual maturity between 2–3 months of age and mate via broadcast spawning into the water column. (**B**) Thus, fertilized embryos can be collected non-invasively and grown in the lab. Embryos develop optimally at a temperature of 28.5 °C, as they are tropical fish, and can be grown in various formats. Here, we show the example of arraying the embryos in multi-well plates to conduct exogenous treatments with small molecules to interrogate developmental processes and for toxicology studies. The use of RA pathway agonists or antagonists, like ATRA or DEAB, can be used to interrogate how changes in RA biosynthesis or signaling affect ontogeny. Such treatments can begin at any timepoint of interest to the researcher, such as the 60% epiboly depicted here, and then embryos reared to the stage(s) of interest, such as 24 hpf, to examine the effects by any number of phenotype assessments, such as live assessment, WISH, or qRT-PCR. Figure created with BioRender.com.

Zebrafish also possess a high fecundity, allowing for genetic screens [134,135,136,137,138,139,140,141,142,143,144] and high throughput assays to assess varied compounds and their role in developmental processes [145,146,147,148,149,150,151,152]. For example, RAR and RXR agonists/antagonists and their role in developmental processes can be explored by adding these compounds at various times (Figure 2). This short generation time paired with high fecundity also allows for large scale forward and reverse genetic screens. Further, in contrast to mammals, zebrafish possess the ability to regenerate many complex tissues and organs such as the kidney, heart, fin, and eye [153,154,155,156,157]. The zebrafish genome has been sequenced and is well annotated, allowing for conservation of biomarkers in health and disease [158]; and there are extensive centralized resources made possible by community efforts [159,160,161]. Genetically, ~70% of human protein coding genes share an ortholog with zebrafish [158]. In comparison to mammalian models, such as mouse and rat, the zebrafish is economically efficient in terms of the space needed to house adequate colonies. The use of zebrafish as a model has become increasingly important for providing insights into development and disease, given the nature and feasibility of many approaches that are unique to the use of this species. For the aforementioned reasons of rapid development, short generation time, and ease of use for screens that may utilize large chemical drug/libraries, zebrafish will continue to be a valuable model for elucidating the roles of RA in development.

## 3. RA in Development

### 3.1. Neural Plate Progenitors

Early hindbrain development is reliant on proper RA signaling. During this process, RA synthesis is thought to be acted on by *pbx2* and *pbx4* to regulate early hindbrain fate decisions [162]. In *aldh1a2*-deficient zebrafish *neckless* (*nls*) mutants [59], the down regulation of *RARɑ* leads to imprecise regulation of *hoxb4.* The neuroectoderm of 14-somite stage *nls* mutants lacks *hoxb4* expression, despite ubiquitous expression in mesodermal tissues [59]. However, by the 16-somite stage (roughly one hour later), *hoxb4* expression is restored to neural tube progenitors within *nls* mutants, thus implying a potential compensatory mechanism of RA-mediated *hoxb4* expression. Both RA and FGF gradients work synergistically to develop positional identities within the rhombomeres, a group of vital cell populations necessary for early neural crest migration [163,164,165,166].

Through the exogenous treatment of embryos with the aldehyde dehydrogenase inhibitor molecule N,N-diethylaminobenzaldehyde (DEAB) (Figure 2) starting at the 4 hpf stage, rhombomere 5 and 6 identities were found to be lost at 16 hpf, implying the importance of RA in signaling fates in early hindbrain populations [166]. Other work performed in zebrafish showed that loss of RAR signaling via treatment with a pan-RAR antagonist led to not only a lack of posterior hindbrain identity, but also an increase in the domain of anterior rhombomeres two, three, and four [61]. This implies that the two tailed gradient of RA is opposed by Cyp26 enzymatic activity at both the far anterior and posterior ends of the developing fish. In work performed by Qiu et al., RA, FGF, and other morphogenic gradients were wonderfully computationally modeled to highlight the precision necessary for essential fates within the developing hindbrain and the associated rhombomeres within zebrafish [167].

In later points of development within the forebrain, loss of both *aldh1a2* and *aldh1a3* results in altered yet intact expression of *fgf8* and *shh*, implying RA is not solely responsible for early forebrain organization in mice [168]. In zebrafish, depletion of *hmx4,* a homeobox gene and ortholog of the human *HMX1*, results in a significant decrease in *aldh1a2* expression which leads to lack of neural tube closure [169]. In these same studies conducted by Gongal et al., the addition of exogenous RA to *hmx4* morphants rescues *gli3* expression and allows for regular forebrain development, suggesting that *hmx4* works via RA to mediate Shh activation [169]. This work opens exciting new avenues for investigation into RA and its role in forebrain development.

Eye development in zebrafish as well as other vertebrates originates within the eye field, a primordial section of the neural plate. Transcriptionally, a grouping of factors known as eye field transcription factors (EFTF) are credited with this specification. These factors include homeodomain genes such as *rx3, otx2,* and *lhx2,* and *pax6* [170]. In RNA-sequencing analysis of *rx3^-/-^* fish at 13 hpf, select RA-related orphan receptors were found to be transcriptionally downregulated [171]. Loss of this EFTF and the resulting down regulation of RA-associated machinery may imply transcriptional priming within these early eye field cells for RA that is used in later regulation of optic development.

### 3.2. Kidney

The nephron is the functional unit in the kidney, and it is tasked with several critical physiological roles: the filtering of the blood through the glomerulus, the facilitation of solutes in and out of circulation via the tubules, and the passage of waste for dismissal in the collecting duct [172]. In higher vertebrates, the pronephros is the first transient form of the kidney, followed by the mesonephros, and the final formation of the kidney is named the metanephros [172]. In zebrafish, only the first two, the pronephros and mesonephros, are formed [173]. Pronephros development in zebrafish begins during the process of intermediate mesoderm formation through the action of signals derived from Bone Morphogenic Proteins (BMPs) [174]. As the intermediate mesoderm forms, RA signals originating from the paraxial mesoderm orchestrate the genesis of the bilateral nephrons [64,175]. These movements can be visualized through the use of transgenic lines expressing renal progenitor field markers such as *lh1xa, pax2a,* and *pax8* [176].

**Figure 3:**
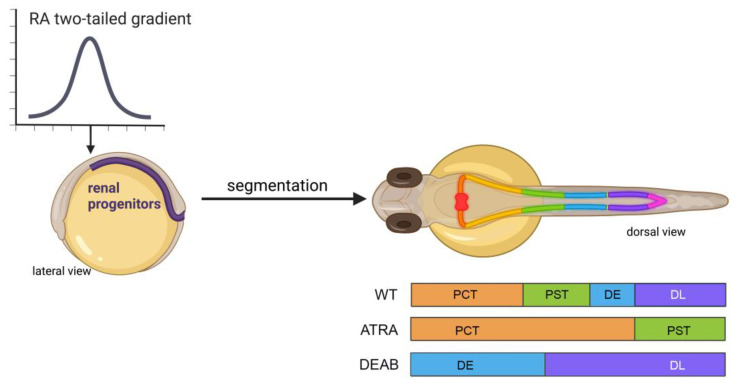
RA levels influence proximo-distal nephron fates during embryonic kidney development in the zebrafish. (**Left**) Renal progenitors are exposed to a gradient of RA during gastrulation and axis formation, with the highest level at the rostral-most locations (arrow). **(Right**, **top**) As development proceeds, these RA levels exert an influence on the proximo-distal segmentation patterning. The zebrafish embryonic kidney comprises two bilateral nephrons with several unique cell populations, and these undergo morphogenesis events by the 48 stage to connect rostrally at a single midline blood filter and caudally at the cloaca where waste exits the body. (**Right**, **bottom**) The effects of RA are best understood for the 4 main tubule populations: the proximal convoluted tubule (PCT), proximal straight tubule (PST), distal early (DE), and distal late (DL). Embryos exposed to a high dosage of ATRA starting at 60% epiboly form nephrons with only proximal segments. Conversely, embryos exposed to the RA biosynthesis inhibitor DEAB starting at 60% epiboly form nephrons with only distal segments. Figure created with BioRender.com and based on [175].

In the presence of exogenous (10^−6^ M) ATRA exposure from 60% epiboly/6 hpf stage to the six-somite stage, two of these same markers, *pax2a* and *pax8,* are found to have the non-discrete spatial expression pattern seen in regular development [175]. In this same line of experiments, use of the RA synthesis inhibitor DEAB at the same six-somite stage results in rostralization of *evi1 (mecom)* [175] a downstream mediator of Fgf signaling [177] and a marker for more distal tubule fates at the 28-somite/24 hpf stage [64,178]. Further analysis of the inhibition of RA synthesis through the comparison of *aldh1a2* knockdown via morpholino oligonucleotide and chemical inhibition via DEAB shows that the time of RA synthesis is crucial to the tubule phenotype within the nephron; and it further confirms the redundancy within ALDH enzymatic expression patterns [175]. In *aldh1a2* knockdown fish, all of the tubules form by 48 hpf; however, distal tubule fates expand rostrally at the cost of anterior segments [175]. In the chemical inhibition of RA synthesis via DEAB, time of addition experiments concluded that RA is most needed in tubule fate decisions between 60% epiboly and 15-somite stages, or approximately 6–16 hpf [175]. Lack of sufficient RA synthesis during this period of gastrulation and somitogenesis leads to the complete loss of proximal cell fates (Figure 3) [175]. This work concluded that RA works to posteriorize proximal cell fates in juxtaposition to Fgf signaling. Though loss of RA is associated with inefficient pronephros formation, overproduction or addition of exogenous RA can be equally detrimental to the organization of the tubules. At the same ATRA concentration (10^−6^ M) used to determine that intermediate mesoderm patterning is sensitive to opposing RA and FGF-inducing gradients, exposure to such high levels of ATRA from 90% epiboly to the 5-somite stage (5–10 hpf) leads to complete loss of distal segment fates and highly increased proximal convoluted tubule (PCT) and proximal straight tubule (PST) populations at the 24 hpf/28-somite stage (Figure 3) [175]. This work, and others involving the nephron and the associated tubule dependence on RA for regulation of segmentation, is well characterized, and the investigation of the downstream gene regulatory network has revealed a number of transcription factors crucial for fashioning segment fates, such as *sim1a*, *tbx2a/b*, and *emx1*, among others [64,179,180,181,182,183,184,185]. However, openings emerge for further interrogation due to the ever-evolving field of RA biology, the ease of performing chemical/drug/biologic screens in the zebrafish model, and the prevalence of next generation sequencing approaches in development.

Within the nephron, a subpopulation of aptly named multiciliated cells (MCCs) project clusters of motile cilia into the tubule lumen and govern fluid flow [186]. MCCs are detectable via WISH as early as the 10-somite stage [186]. Much like the nephron tubule populations, MCCs are dependent on RA signaling for differentiation from the renal progenitors [178]. In the proposed MCC regulatory network, RA works upstream to downregulate *mecom*, a positive regulator of Notch, and a factor for distal cell fates [178]. In knockdown of *mecom* and the subsequent addition of DEAB from late gastrulation to 24 hpf, MCC formation was almost completely rescued in terms of the domain they occupied within the nephrons and the position of this domain in the developing fish [178]. These experiments involving RA signaling and MCC fate determinants highlight exciting avenues of future interrogation into the potential regenerative capacities of MCCs after injury, which may lend potential insights into human disease.

### 3.3. Heart

From its genesis, the zebrafish heart is reliant on RA for correct spatial patterning of progenitors. Work performed by Keegan et al. delicately described both RA addition and the time in which RA is necessary for restricting progenitor fates [187]. When they treated embryos with a pan-RAR antagonist and then assessed *cmlc2* and *nkx2.5* expression, which marking cardiomyocytes precardiac mesoderm, respectively, they observed increased domains of expression at the 16-somite stage, suggesting RA works in restricting cardiac cell fates in post gastrulation fish [187]. This hypothesis was then confirmed through the use of exogenous ATRA treatments spanning a time point straddling gastrulation initiation leads to lessened *cmlc2* expression at the 18-somite stage [187]. The mechanism of RA restricting the cardiac field was further characterized as being mediated by *cdx4* and *cdx1a* expression. In this process, much like in hindbrain development, RA works through downregulating *cdx4* expression, thereby activating *cyp26a1* expression to metabolize RA within the cell [188,189]. This hypothesis further postulates the intricate nature of RA self-regulation, as RAREs exist as regulatory units of *cyp26/Cyp26/CYP26* family expression [116,117]. Other work involving regulators of cardiac cell fate such as *NR2F* have been found to have RARES within these factors’ promoter regions, which in the presence of DEAB leads to a decreased *nr2f1a* domain. The loss of *nr2f1a/b* contributes to a relative expansion of the *nkx2.5* domain, a commonality seen with RAR antagonism.

Further investigation into RA and its role in early heart field regulation revealed that RA works indirectly through the homeobox gene *hoxb5b* to limit cardiac progenitor populations [190]. Other work established that RA and FGF signaling worked in opposing manners to regulate heart field establishment, as the heat shock resulting in overexpression of FGF machinery in late gastrulation led to increased cardiac cell count, a phenotype also seen in DEAB-treated embryos [191,192]. Similarly, *Aldh2* null mouse embryos bear a posteriorization and general domain increase of *Fgf8* and its target *Isl1* within the developing cardiac field, thus leading to improper chamber development [193,194].

RA signaling-associated machinery has also been a topic that has provided insights into RA and its role in heart development. One avenue of investigation included that of Cyp26 metabolic action for its role in maintaining RA equilibria. Opposite of a pan-RAR antagonist, Cyp26-deficient embryos result in a decreased heart field domain marked by *nkx2.5,* in course leading to decreased ventricular fates later in development [195]. Interestingly, the use of DEAB on these same *cyp26* morphants results in a restoration of expression, concluding that loss of RA in early cardiac development results in increased heart field size, while increases in RA restrict these early progenitor populations and can alter later fate decisions [195]. These insights into RA’s role in cardiac development not only provide an understanding of base developmental processes, but also present a translational model for in utero VAD and accompanying congenital defects of the heart.

In work performed at later stages in development, Cyp26-deficient embryos were found to have increased *mmp9* expression, leading to improper outflow tract morphogenesis due to the diminished addition from second heart field progenitors [196]. This work proposed a potential mechanism for RA-mediated outflow tract defects, and adds to the body of knowledge involving the relationship between vitamin A and *mmp9*. Further avenues of interrogation into processes associated with retinoid transport and synthesis within cardiac/cardiac progenitor cells remain open for insights, and make for exciting new lines of investigation.

One of the many reasons the zebrafish is such an attractive model is due to its regenerative capacity within the heart and other tissues [197]. In the ventricular amputation model [198], one hour after ventricular amputation of the adult zebrafish heart, *aldh1a2* expression was remarkably abundant within the atrial endocardium, and within 3 days post amputation (dpa), expression of the RA-synthesizing enzyme was also localized to the epicardium, then to the epicardial cells that surround the wound clot by 7 dpa [199,200].

In this same line of experiments performed by Kikuchi et al., overexpression of *cyp26a1*, the enzyme responsible for RA degradation, led to dramatic decreases in cardiomyocyte proliferation at seven days post injury [200]. However, in the case of retinoid agonist supplementation, no increase in cardiomyocyte proliferation was induced, thus inferring that RA does not promote the regenerative process, but rather plays a permissive role [200]. Within the mouse model of acute damage to the heart, conflicting reports surround RA, and the protective effects it may or may not have in the case of myocardial infarction-induced cellular death [201,202].

### 3.4. Hematopoiesis

Research with the zebrafish has provided many fundamental insights into the genetic mechanisms of primitive and definitive hematopoiesis [203], from events involving hematopoietic stem cell (HSC) patterning [204] to fate choice [205,206] and differentiation [207,208,209,210]. A number of studies have contributed new insights into the effects of RA signaling on both the primitive and definitive hematopoietic waves, which are distinct in the cells they produce; namely, red blood cells and macrophages during the initial wave, followed by the production of all lineages in the subsequent wave, respectively [203].

Exogenous ATRA treatment of embryos from the late gastrula to the 5-somite stage elicits a dose-dependent inhibition of primitive erythropoiesis, based on reduced expression of the erythroid-specific transcription factor *gata1* [211], and inhibition of primitive myelopoiesis, based on reduced expression of several markers [212]. Conversely, DEAB treatment increased specification of hematopoietic stem and progenitor cells, leading to elevated primitive erythropoiesis [213]. Further, interference in RA signaling with DEAB or though *aldh1a2* knockdown has been associated with reductions in definitive blood cell production based on reduced expression of stem cell markers such as *cmyb* and thymic *rag1/ikaros* expression [214]. These findings parallel the ability of RA to enhance progenitor formation in culture [215], and the requirement for Aldh1a2 expression in the endothelium to produce definitive HSCs [216].

### 3.5. Pancreas, Liver, and Intestine

Within the endoderm, several organs/tissues have been characterized to have RA-dependent morphogenesis. Within the developing liver, RA positively regulates liver fates, as exogenous treatment of RA during varied significant points during hepatic differentiation led to increased expression of *fabp1a* (*lfabp)* at 72 hpf, while DEAB treatment at like times results in decreased *fabp1a^+^ domain* [217]. In this same line, fish treated with RA show increased levels of proliferation within the liver primordium via BrdU at 24 hpf [217]. Further investigation into the RA pathway by Garnaas et al. showcased *rargb* as being a regulator of early heart and liver morphogenesis, as *rargb* morphants and antagonists led to aberrant phenotypes in both tissues, falling in line with RAR agonist work involving cardiac cell proliferation [217,218]. In the developing intestines, decreases in RA biosynthesis, specifically in retinol dehydrogenase expression, were found to restrict gut marker expression domains in 96 hpf fish [219,220]. In these same experiments, interestingly, exogenous treatment of RA was found to only partially rescue the *rdh1l* morphants [220]. These results postulate that RA is a positive mediation of these two foregut tissues. These findings also suggest exciting avenues and niches for discovery within the realm of retinoid biology, especially with regard to downstream targets of R(A/X)Rs.

In the mouse model, *aldh1a2* null animals were found to lack dorsal pancreatic tissues [221]. In fish, *aldh1a2* null animals do not retain this same phenotype, as *pdx1*, a marker for beta cell progenitor populations was found to be expressed at 48 hpf, and other pancreatic markers were expressed at 72 hpf [63,222]. Further work utilizing both splice blocking and ATG-binding morpholinos, as well as DEAB, suggested that *aldh1a2* is maternally deposited within fish due to DEAB and ATG-binding morphants resulting in the loss of pancreatic marker expression; meanwhile, *aldh1a2* null and splice blocking MO-treated fish retained a domain, however aberrant [222]. During pancreatic development, loss of RA signaling via RAR pan-antagonist BMS493 before late gastrulation (90% epiboly) results in lack of *insulin* expression [223,224,225,226]. Conversely, ectopic ATRA (10^−6^ M treated from 9–10 hpf) results in the upregulation of *insulin* expression within 24 hpf animals, as well as increasing the *insulin^+^* domain anteriorly, thus furthering the idea of RA-mediated beta cell proliferation across multiple models [223,224,225,226].

Recently, exciting new avenues thanks to multi-omic data have been revealed, and have opened for further investigation in RA/hepatopancreatic/endodermal biology fields. Insights into partially retained machinery across all three germ layers within the zebrafish were established. Among these observations, it was particularly interesting that *hox* clusters were established as being hosts to RAR sites, as this helps to confirm the idea of *hox* regulation of endodermal foregut tissues in the zebrafish model [227]. These same studies established RARs as mediating the expression of pioneer factors such as *hnf1b and gata6* within the pancreas [227].

## 4. RA in Disease and Dysfunction

### 4.1. RA in Deficiency and Surplus in Humans

**Figure 4:**
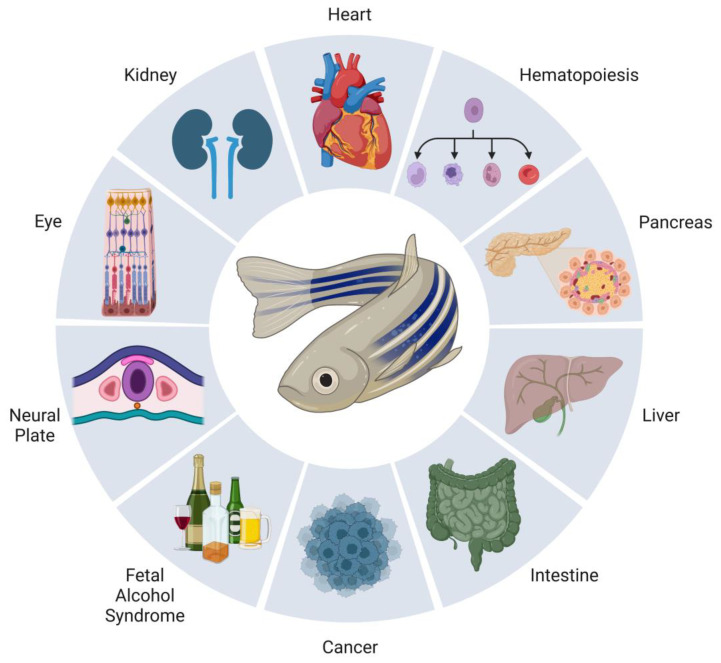
Summary of zebrafish models of RA signaling in development and disease. Research using the zebrafish has been a powerful good for realizing new insights into the development of many tissues and organs. Numbering among them, and discussed in the present work, are the neural plate, eye, kidney, heart, blood, pancreas, liver, and intestine. Many others have been studied as well [17]. Our understanding about the teratogenic effects of RA in conditions such as fetal alcohol syndrome and its role in cancer have also been expanded through the use of zebrafish. Figure created with BioRender.com [232].

In addition to studying development, zebrafish have been a powerful system to study human disease (Figure 4) [18,19]. In work utilizing zebrafish, loss of RA associated with exposure to teratogenic compounds such as alcohol has been well documented. Zebrafish as a model for fetal alcohol syndrome has been attractive for the many reasons previously described [228,229]. In fish, when treated with 150 mM of ethanol from 3–24 hpf, severe phenotypes were observed within tissues and organs such as the eyes, otic vesicle, facial cartilage, and pericardial edema, among others [230]. These malformations fall in line with the severe defects seen clinically, as eye, heart, and improper craniofacial development are all found. These maladies are thought to be due to acetaldehyde, the byproduct of ethanol metabolism further being metabolized by an ALDH, thus reducing retinaldehydes’ ability to bind to ALDHs. In order to bypass this inhibition of RA synthesis, exogenous ATRA treatments at low levels (10^−9^ M) have been found to rescue the effects of ethanol exposure [230,231].

Much like insufficient RA is detrimental for fetal development in humans, an excess can be equally as harmful. Perhaps most notably, the dermatological drug isotretinoin, an RA isomer, is well characterized for the teratologic role it can have in pregnant individuals and their offspring. In utero exposure to the drug is estimated to have a 20–35% chance of congenital defects to many of the same tissues we have described as being RA-dependent in development. In terms of immeasurable maladies, it is estimated that potentially over 50% of individuals exposed to isotretinoin in utero may have cognitive impairment [233,234]. In zebrafish, isotretinoin has been used in neuroblastoma work, and has been successfully shown to have the capacity for decreasing tumor size in larval fish [235].

### 4.2. RA Pathways in Cancer

Within the realm of cancer biology, the zebrafish model has emerged as an efficient in vivo model, much for the same reasons why it is so popular in development. Thankfully, due to high amounts of conservation between the human and zebrafish genome, many oncologic markers are subsequently retained, making the fish useful for studying many aspects of cancer biology (Figure 4) [158,236,237]. The zebrafish immune system allows for tissue xenografts from mammalian donors, allowing for not only large-scale drug and genetic screens to take place, but also holding promise for a future in precision medicine [238,239,240,241].

In a zebrafish pancreatic cancer model, antagonization of RARs were used to downregulate miR-10a expression, resulting in a loss of invasive and metastatic phenotype; interestingly, it was also found that microRNA-10a (miR-10a) is an intermediate regulator between RARs and *hox1* and *hoxb3* [242]. This same work also showed that miR-10a is also upregulated in pancreatitis patient samples; this postulates the questions of whether RA is mediating the micro-RNA in both disease states, and if RA regulation of miR-10a is associated with normal development of the pancreas [242]. 

In neuroblastoma, loss of chromatin assembly factor 1 subunit p150 (CHAF1A) promotes oncogenesis/malignancy [243]. Alongside a mouse model, zebrafish were used to investigate *chaf1a* expression within regular neural crest cell development as well as carcinogenesis [244]. The use of ectopic *chaf1a* expression revealed that *chaf1a* plays roles in the critical fate determinant stages of neural crest cells in development. This work was in step with mouse work that was then used to hypothesize that RA could be used in combinatorial therapies for those diagnosed with neuroblastomas [244]. In the future, we believe zebrafish will further emerge as an in vivo model for oncological work as a supplement for early genetic and/or pharmacological screening methods, as well as for targeting therapeutics for individual patients [245].

## 5. Conclusions

Here, we have discussed how zebrafish research has been utilized to uncover new fundamental insights about the roles of RA signaling in the development of several tissues and organs, as well as in regeneration and disease. Continuing technological advances in multi-omics, combined with the tractability of zebrafish for pharmacological treatments, have facilitated these advances, and offer many prospects for future studies.

Our knowledge about the molecular targets of RA remains in its infancy, and more work is needed to decipher these gene regulatory networks, and to understand how the respective targets act to confer RA’s effects on cell fate specification and differentiation. Appreciating these facets may uncover new prospects for retinoids in clinical therapeutics. Indeed, the next century will be an exciting time in RA research, and the zebrafish will continue to proffer unique opportunities to narrow our knowledge gap about the many roles of this versatile and potent molecule in living systems.

## Data Availability

Not applicable.
